# Infectious Etiologies of Chronic Diseases: Focus on Women^1^

**DOI:** 10.3201/eid1011.040623_07

**Published:** 2004-11

**Authors:** Siobhán O'Connor, DeLisa Fairweather, Brad D. Pearce, Sonja Rasmussen

**Affiliations:** * Centers for Disease Control and Prevention, Atlanta, Georgia, USA;; † Johns Hopkins University, Baltimore, Maryland, USA;; ‡ Emory University School of Medicine, Atlanta, Georgia, USA

**Keywords:** Infectious etiologies of chronic disease, chronic disease, infection, women, autoimmune, neurodevelopmental disorder

Infections can directly or indirectly cause chronic conditions through progressive pathology (e.g., chronic infection, inflammation, immunity, malignant transformation), sudden permanent insults (e.g., West Nile virus poliomyelitis paralysis), or by predisposing people to noninfectious sequelae (e.g., neurologic consequences of preterm birth). Bacteria, parasites, prions, viruses, and fungi may be the single or one of several factors contributing to chronic disease; one organism can cause more than one syndrome, and diverse pathogens produce similar syndromes as pathways to disease converge (*1*). Certain potential outcomes disproportionately affect women (e.g., autoimmune diseases), and in some settings, detection, prevention, or treatment efforts (e.g., ocular trachoma, underdiagnosed genital infections) may marginalize women. Women's activities can also increase exposures to chronic disease pathogens (e.g., schistosomiasis attributable to chores or agriculture), and gender can affect transmission (e.g., increased male-to-female transmission of human T-cell leukemia virus–1). Preventing maternal infections may further minimize chronic disease and neurodevelopmental disorders in offspring.

## Are Women's Autoimmune Diseases Really Autoimmune?

Systemic and organ-specific autoimmune diseases, such as rheumatoid arthritis and myocarditis, are the leading cause of death in women >65 years of age (*2*). They affect 14–22 million people (5%–8% of the population) in the United States (*3*) and millions more worldwide. In autoimmunity, the immune system may attack or damage self-tissues with autoantibodies and autoreactive T and B cells. However, the indolent nature of most autoimmune diseases makes determining infectious triggers difficult. Animal models help to understand such links. For example, transfer of disease by autoantibodies and immune cells from affected animals indicates the immune-mediated nature of these syndromes (*4**–**6*). Toll-like receptors and the innate immune system, critical components of the normal human response to infection, are essential to naturally and experimentally induced autoimmunity. Genetic and other factors affect susceptibility to both infection and autoimmune disease. For example, coxsackievirus B3 induces viral myocarditis in susceptible mice. Certain cytokines (interleukin [IL]-1β and tumor necrosis factor [TNF]-α), but not viral replication, correlate with cardiac inflammation and can overcome resistance to chronic myocarditis (*7**–**9*). These findings suggest that, while infection may trigger autoimmunity, immune processes drive disease progression. Estrogen amplifies the immune response to coxsackievirus B3 in susceptible mice, increasing TNF-α and IL-4 levels (unpub. data), which is perhaps consistent with women's predisposition to autoimmune disease. Identifying triggers, including infection, and early markers of autoimmunity are important goals for preventing onset of or disrupting progression to autoimmune disease.

## Infection Connection in Neurodevelopmental Disorders

Intrauterine infections are known causes of congenital defects worldwide. Infections during the time of fetal brain development might also contribute to neuropsychiatric disorders, including schizophrenia. Studies linking various gestational insults (including infections) and subtle premorbid behavioral alterations to adult schizophrenia implicate a neurodevelopmental origin. However, the long latency between putative infection or insult and the emergence of psychotic symptoms complicates establishing direct links. While most reports have been ecologic studies without confirmed maternal infection, Brown et al. (*10*) found that 20.4% of persons with a documented in utero exposure to rubella developed an adult schizophrenia spectrum disorder. Experimentally, lymphocytic choriomeningitis virus infection in a neonatal rat model produces some latent changes similar to those of schizophrenia, e.g., hippocampal atrophy and impaired inhibitory GABA neurotransmission (*11*); blocking IL-1 partially attenuates the hippocampal cell loss. Inflammatory cytokine responses, perhaps amplified by immunogenetic abnormalities, may be a common thread linking intrapartum infections and noninfectious gestational and obstetric complications to neurodevelopmental disorders (*12*).

## Keys to the Future

A continuum from acute infection to chronic disease exists, and each stage is an opportunity to prevent or minimize an avoidable fraction of chronic disease—that resulting from infectious disease. Crucial steps include identifying infectious etiologies and cofactors, determining persons (including women) at risk for infection or outcome, and implementing measures that minimize chronic sequelae. Research incorporating longitudinal studies that precede clinical disease must support evidenced-based conclusions and actions. The benefits to women could be substantial.
